# Genetic diversity and genomic epidemiology of SARS-CoV-2 during the first 3 years of the pandemic in Morocco: comprehensive sequence analysis, including the unique lineage B.1.528 in Morocco

**DOI:** 10.1099/acmi.0.000853.v4

**Published:** 2024-10-07

**Authors:** Soulandi Djorwé, Abderrahim Malki, Néhémie Nzoyikorera, Joseph Nyandwi, Samuel Privat Zebsoubo, Kawthar Bellamine, Amale Bousfiha

**Affiliations:** 1 Laboratory of Physiopathology and Molecular Genetics, Faculty of Sciences Ben M'Sik, Hassan II University of Casablanca (Morocco), Avenue Cdt Driss El Harti, PB 7955 Sidi Othman, Casablanca, Morocco; 2 Bourgogne Laboratory of Medical and Scientific Analysis, 136, residence belhcen, Bd Bourgogne, Casablanca, Morocco; 3 National Reference Laboratory, National Institute of Public Health, Bujumbura, Burundi; 4 Higher Institute of Biosciences and Biotechnology, Mohammed VI University of Health Sciences (UM6SS), Casablanca, Morocco; 5 Laboratory of Microbial Biotechnology and Infectiology Research, Mohammed VI Center for Research & Innovation, Mohammed VI University of Health Sciences (UM6SS), Casablanca, Morocco; 6 Département de Médecine, Faculté de Médecine, Université du Burundi, Bujumbura, Burundi; 7 Ministère de la Santé Publique et de la Lutte contre le Sida, Institut National de Santé Publique de Bujumbura, Bujumbura, Burundi; 8 School of Advanced Studies in Biotechnology and Private Health (EHEB), 183 Bd de la Resistance, Casablanca 20250, Morocco

**Keywords:** genetic diversity, genomic sequence, lineage, omicron, SARS-CoV-2, variant

## Abstract

During the 3 years following the emergence of the COVID-19 pandemic, the African continent, like other regions of the world, was substantially impacted by COVID-19. In Morocco, the COVID-19 pandemic has been marked by the emergence and spread of several SARS-CoV-2 variants, leading to a substantial increase in the incidence of infections and deaths. Nevertheless, the comprehensive understanding of the genetic diversity, evolution, and epidemiology of several viral lineages remained limited in Morocco. This study sought to deepen the understanding of the genomic epidemiology of SARS-CoV-2 through a retrospective analysis. The main objective of this study was to analyse the genetic diversity of SARS-CoV-2 and identify distinct lineages, as well as assess their evolution during the pandemic in Morocco, using genomic epidemiology approaches. Furthermore, several key mutations in the functional proteins across different viral lineages were highlighted along with an analysis of the genetic relationships amongst these strains to better understand their evolutionary pathways. A total of 2274 genomic sequences of SARS-CoV-2 isolated in Morocco during the period of 2020 to 2023, were extracted from the GISAID EpiCoV database and subjected to analysis. Lineages and clades were classified according to the nomenclature of GISAID, Nextstrain, and Pangolin. The study was conducted and reported in accordance with STROBE (Strengthening the Reporting of Observational Studies in Epidemiology) guidelines. An exhaustive analysis of 2274 genomic sequences led to the identification of 157 PANGO lineages, including notable lineages such as B.1, B.1.1, B.1.528, and B.1.177, as well as variants such as B.1.1.7, B.1.621, B.1.525, B.1.351, B.1.617.1, B.1.617.2, and its notable sublineages AY.33, AY.72, AY.112, AY.121 that evolved over time before being supplanted by Omicron in December 2021. Among the 2274 sequences analysed, Omicron and its subvariants had a prevalence of 59.5%. The most predominant clades were 21K, 21L, and 22B, which are respectively related phylogenetically to BA.1, BA.2, and BA.5. In June 2022, Morocco rapidly observed a recrudescence of cases of infection, with the emergence and concurrent coexistence of subvariants from clade 22B such as BA.5.2.20, BA.5, BA.5.1, BA.5.2.1, and BF.5, supplanting the subvariants BA.1 (clade display 21K) and BA.2 (clade display 21L), which became marginal. However, XBB (clade 22F) and its progeny such XBB.1.5(23A), XBB.1.16(23B), CH.1.1(23C), XBB.1.9(23D), XBB.2.3(23E), EG.5.1(23F), and XBB.1.5.70(23G) have evolved sporadically. Furthermore, several notable mutations, such as H69del/V70del, G142D, K417N, T478K, E484K, E484A, L452R, F486P, N501Y, Q613H, D614G, and P681H/R, have been identified. Some of these SARS-CoV-2 mutations are known to be involved in increasing transmissibility, virulence, and antibody escape. This study has identified several distinct lineages and mutations involved in the genetic diversity of Moroccan isolates, as well as the analysis of their evolutionary trends. These findings provide a robust basis for better understanding the distinct mutations and their roles in the variation of transmissibility, pathogenicity, and antigenicity (immune evasion/reinfection). Furthermore, the noteworthy number of distinct lineages identified in Morocco highlights the importance of maintaining continuous surveillance of COVID-19. Moreover, expanding vaccination coverage would also help protect patients against more severe clinical disease.

## Data Summary

The sequence data used in this investigation were extracted from the GISAID EpiCov database. All the sequences analysed in this study are available under the accession number (GISAID identifier): EPI_SET_240225ry (File S1, available in the online version of this article). Supplementary Material is provided alongside the article in PDF and Excel formats. Access to the Supplementary Material is available on Figshare via the following DOI link: https://doi.org/10.6084/m9.figshare.25748970.v1 [1].

## Availability of Data

The results presented in this study are derived from the analysis of 2274 genomic sequences of metadata available on: GISAID Identifier: EPI_SET_240225ry. DOI: 10.55876/gis8.240225ry.

## Introduction

Appearing in 2019 in Wuhan, in the Hubei province, China, COVID-19 is one of the most devastating pandemics of this century, involving millions of cases and deaths worldwide. In February 2020, the first case of COVID-19 was reported in Africa, in Egypt, and the first case in Morocco was reported on 2 March 2020 [2]. Moreover, in Morocco, a national SARS-CoV-2 genomic surveillance consortium has been set up to sequence Moroccan isolates in order to inform the health authorities about circulating variants. As of 6 August 2023, more than 769 million cases of infection and over 6.9 million deaths have been reported worldwide, according to the World Health Organization (WHO) [3]. In Morocco, during the last quarter of 2023, over 1  278  055 confirmed cases and 16  298 deaths were recorded [4].

The SARS-CoV-2, an RNA virus of the coronavirus family responsible for the global COVID-19 pandemic, has a genome length of approximately 29.9 kilobases (kb) [5]. The genome consists of 15 ORFs, with two important polyproteins involved in replication: pp1a and pp1ab (ORF1ab). These polyproteins are cleaved to produce 16 non-structural proteins. Additionally, the genome codes for 11 accessory proteins and four structural proteins, including the Spike glycoprotein, envelope, membrane, and nucleocapsid proteins [5,7]. Generally, within the SARS-CoV-2 genome, mutations can progressively accumulate over time, in particular, key mutations in structural proteins, of which the Spike glycoprotein is the most closely monitored. Moreover, some mutations in the SARS-CoV-2 genome can significantly impact its fundamental properties. These mutations can increase contagiousness, enhance the severity and virulence of the infection, reduce the effectiveness of drugs and vaccines, allow the virus to evade the immune system, cause reinfections, and escape molecular detection during laboratory diagnostics. Therefore, variants that exhibited the aforementioned characteristics are classified either as variants of concern (VOC), either as variants under monitoring (VUM) or variants of interest (VOI). However, these variants required close monitoring [8,9].

This study aimed to retrospectively analyse data from sequences derived from Moroccan isolates during the COVID-19 pandemic, in order to better understand the genomic epidemiology of SARS-CoV-2 in Morocco. The main aim of this study was to analyse the genetic diversity of SARS-CoV-2 and identify distinct lineages, as well as to assess their evolution during the pandemic in Morocco, using genomic epidemiology approaches. Furthermore, several key mutations in the functional proteins across different viral lineages were highlighted along with an analysis of the genetic relationships amongst these strains to better understand their evolutionary pathways.

The study conducted will provide access to in-depth data analysis with detailed large-scale analysis. Furthermore, this study will also shed light on the genetic trajectory of SARS-CoV-2, specifically the appearance and distribution of variants during the 3 years after the pandemic’s onset in Morocco. This study will extend our understanding of the elements affecting the genetic variation of SARS-CoV-2 in Morocco, such as mutation rates and transmission dynamics. Finally, it will provide a strong basis for understanding the virus’s evolution, facilitating the development of more precise preventive measures in future research. However, expanding the analysis by incorporating global GISAID sequences would contribute to a better development of preventative measures.

## Methods

### Study design

This study was conducted and reported in accordance with STROBE (Strengthening the Reporting of Observational Studies in Epidemiology) guidelines [10].

### Sequence data acquisition

The complete genomic sequences of SARS-CoV-2 isolates, collected in Morocco from 2020 to 2023, were extracted in FASTA format from the GISAID EpiCoV database (https://gisaid.org/, accessed on 23 January 2024) [11]. The genomic sequences of the Moroccan isolates were compared with the Wuhan-Hu-1 reference genome, identified by accession number NC_045512.2 in the GenBank database.

File S1 contains the digital object identifier (DOI) and EPI_SET identifier of the 2274 SARS-CoV-2 genomic sequences used in this study. The collection dates range from 2 February 2020 to 3 November 2023.

### Sequence alignment and phylogenetic analysis of Moroccan genomes

In this study, we used standard dynamic classification systems to assign genetic lineages and viral clades. The classification of genomic sequences into lineages was achieved by Pangolin COVID-19 lineage assigner version 4.3, which is a Phylogenetic Assignment tool for Named Global Outbreak LINeages developed by the Centre for Genomic Pathogen Surveillance (https://cov-lineages.org/resources/pangolin.html, accessed on 23 January 2024) and/or Nextstrain web tool version 3.3.1 (https://clades.nextstrain.org/, accessed on 23 January 2024) [12,14]. Rigorous quality checks and the assignment of viral clades were performed using the Nextclade Web and GISAID. The phylogenetic tree was generated using the UCSC UShER Web interface (https://genome.ucsc.edu/util.html, accessed on 25 January 2024), Microreact (https://microreact.org/upload, accessed on 3 February 2024), and Nextclade. Viral clades were defined based on shared mutation profiles among the analysed genomic sequences [14,15].

### Analysis of mutation profiles and assignment of lineages and clades

The GISAID database, the Nextclade and Coronapp web tool (http://giorgilab.unibo.it/coronannotator/, accessed on 20 February 2024) [16] were used to detect and annotate all mutations, thus establishing the single nucleotide polymorphism (SNP) profile of the 2274 genomic sequences. This was achieved by identifying substitutions (amino acid), deletions, or insertions (Indels) in structural protein regions, as well as in some regions of non-structural protein (ORF1ab) [NSP1 to NSP16]. Furthermore, several international reference tools and platforms were used to assign SARS-CoV-2 genomic lineages and clades. The GISAID platform was used to assign lineages and clades. Nextclade Web Tool was used to align sequences and identify specific mutations in comparison with the Wuhan-Hu-1 reference sequence, as well as for the phylogenetic placement of lineages. In addition, Nextclade was also used to assign sequences to lineages and clades according to their specific mutational characteristics. Pangolin was used to assign SARS-CoV-2 lineages according to the PANGO nomenclature and is available both as a web application and as a command-line tool on « Cov-Lineages ». Consequently, these integrated approaches provided a detailed analysis of the 2274 genomic sequences, including their phylogenetic placement and assignment to clades [11,13, 14].

## Results

### Genomic diversity and demographic distribution of SARS-CoV-2 sequences

A set of 2274 genomic sequences of SARS-CoV-2 collected in Morocco over the 3 years following the pandemic has been analysed, revealing several variants and lineages. Table 1 shows the temporal distribution of variants and lineages among the 2274 sequences analysed. Of the 2274 sequences, 3.9%(89/2274) of isolates were sequenced in 2020, 22.4%(511/2274) in 2021, 53.5%(1217/2274) in 2022, and 20.1%(457/2274) in 2023. Among the 2274 sequences analysed, 20.2%(460/2274) were assigned to lineages other than the Alpha, Beta, Delta, Eta, Kappa, Mu, and Omicron variants. Of the 460 sequences analysed, 19.3%(89/460) were identified in 2020, 40%(184/460) in 2021, 4.1%(19/460) in 2022, and 36.5%(168/460) in 2023. The Alpha variant had a prevalence of 7.7%(176/2274) among all sequences analysed, of which 81.2%(143/176) of sequences were detected in 2021, 5.1%(9/176) in 2022, and 13.6%(24/176) in 2023. The Delta variant and its subvariants accounted for 11.3%(257/2274) of the analysed sequences, among which 62.6%(161/257) were identified in 2021, 28.4%(73/257) in 2022, and 8.9%(23/257) in 2023. The Omicron variant and its subvariants were identified in 59.5%(1353/2274) of all sequences analysed, of which 1.1%(15/1353) were identified in 2021, 82.3%(1114/1353) in 2022, and 16.5%(224/1353) in 2023 (Table 1). Furthermore, the other variants such as Beta, Eta, Kappa, and Mu were less predominant. These findings illustrate the dynamic evolution of SARS-CoV-2 variants and lineages during the pandemic, highlighting periods of predominance of variants and lineages identified over time.

**Table 1.** Temporal distribution of SARS-CoV-2 variants and lineages among the 2274 sequences analysed: annual and cumulative prevalence of variants and lineages identified from 2020 to 2023

Variants’year	Alpha	Beta	Delta	Eta	Kappa	Mu	Omicron	Other lineages	Total
2020	0	0	0	0	0	0	0	19.3%(89/460)	3.9%(89/2274)
2021	81.2%(143/176)	16.6%(1/6)	62.6%(161/257)	57.1%(4/7)	100%(1/1)	14.3%(2/14)	1.1%(15/1353)	40%(184/460)	22.4%(511/2274)
2022	5.1%(9/176)	16.6%(1/6)	28.4%(73/257)	14.2%(1/7)	0	0	82.3%(1114/1353)	4.1%(19/460)	53.5%(1217/2274)
2023	13.6%(24/176)	66.6%(4/6)	8.9%(23/257)	28.5%(2/7)	0	85.7%(12/14)	16.5%(224/1353)	36.5%(168/460)	20.1%(457/2274)
Total	176	6	257	7	1	14	1353	460	2274

Of the 2274 sequences analysed, 54.7%(1244/2274) were recorded in the Casablanca region, followed by 15.1%(343/2274) in Rabat, 1.3%(30/2274) in Ouarzazate, 1.2%(27/2274) in Fès, 1%(21/2274) in Mohammedia, 0.7%(17/2274) in Marrakech, while the remaining 26%(592/2274) were from other cities in Morocco.

### Frequency distribution of the 2274 SARS-CoV-2 sequences into lineages

Table 2 shows the frequencies of variants and lineages according to their dominance among the 2274 genomic sequences analysed, and their classification in the clades defined by GISAID and Nextstrain. Among these sequences, ten clades were identified according to GISAID nomenclature and 30 based on Nextstrain classification. The GRA (Omicron) clade had a prevalence of 59.5%(1353/2274) of all sequences analysed, making it the predominant clade in this study. Other lineages, such as B, B.1, B.1.1, B.1.528, B.1.258, B.1.177, B.1.1.487, B.1.1.378, and their sublineages, represented 20.23%(460/2274) of the sequences, distributed in GISAID clades G-GH-GR-GV-S-V. Furthermore, the GK clade (Delta) was mainly composed of the B.1.617.2 and its sublineages (AY.*) with a frequency of 11.30%, followed by the GRY clade (Alpha), dominated by the B.1.1.7 variant, with a prevalence of 7.74%. On the other hand, variants such as Beta, Eta, Mu, and Kappa had frequencies below 1% (Table 2).

**Table 2.** Frequencies of variants and lineages among the 2274 genomic sequences analysed, including the assignment of GISAID and Nextstrain clades for each variant or lineage

WHO nomenclature (lineage)	Clade_GISAID	Clade_Nextstrain	No. of sequences	Frequency (%)
Omicron (B.1.1.529/BA. */BQ.1/BQ.1. */BQ1.1)	GRA	21K-21L-22A-22B-22C-22D-22E	1246/2274	54.79%
Unassigned name (B, B.1, B.1.1, B.1.528, B.1.258, B.1.177, B.1.1.487, B.1.1.378 and other lineages)	G-GH-GR-GV-S-V	19A-19B-20A-20B-20C-20D-20E	460/2274	20.23%
Delta (B.1.617.2/AY. *)	GK	21A-21I-21J	257/2274	11.30%
Alpha (B.1.1.7)	GRY	20I	176/2274	7.74%
Omicron (XBB.1.9.1/XBB.1.9.1. *)	GRA	23D	37/2274	1.63%
Omicron (XBB.1.5/XBB.1.5. *)	GRA	23A-23G	25/2274	1.10%
Omicron (XBB/XBB. * Excluding XBB.1.5, XBB.1.16, XBB.1.9.1, XBB.1.9.2 and XBB.2.3)	GRA	22F	20/2274	0.88%
Mu (B.1.621)	GH	21 H	14/2274	0.62%
Omicron (XBB.2.3/XBB.2.3. *)	GRA	23E	8/2274	0.35%
Eta (B.1.525)	G/484K.V3	21D	7/2274	0.31%
Omicron (XBB.1.16/XBB.1.16. *)	GRA	23B	7/2274	0.31%
Beta (B.1.351)	GH/501Y.V2	20 H	6/2274	0.26%
Omicron (BA.2.75/BA.2.75. *)	GRA	22D	4/2274	0.18%
Omicron (XBB.1.9.2/XBB.1.9.2. *)	GRA	23D	3/2274	0.13%
Omicron (EG.5/EG.5. *)	GRA	23F	2/2274	0.09%
Kappa (B.1.617.1)	G/452 R.V3	21B	1/2274	0.04%
Omicron (GP.2/CH.1.1/CH.1.1. *)	GRA	23C	1/2274	0.04%

*Indicates sublineage (s) or subvariant (s).

**Fig. 1.**
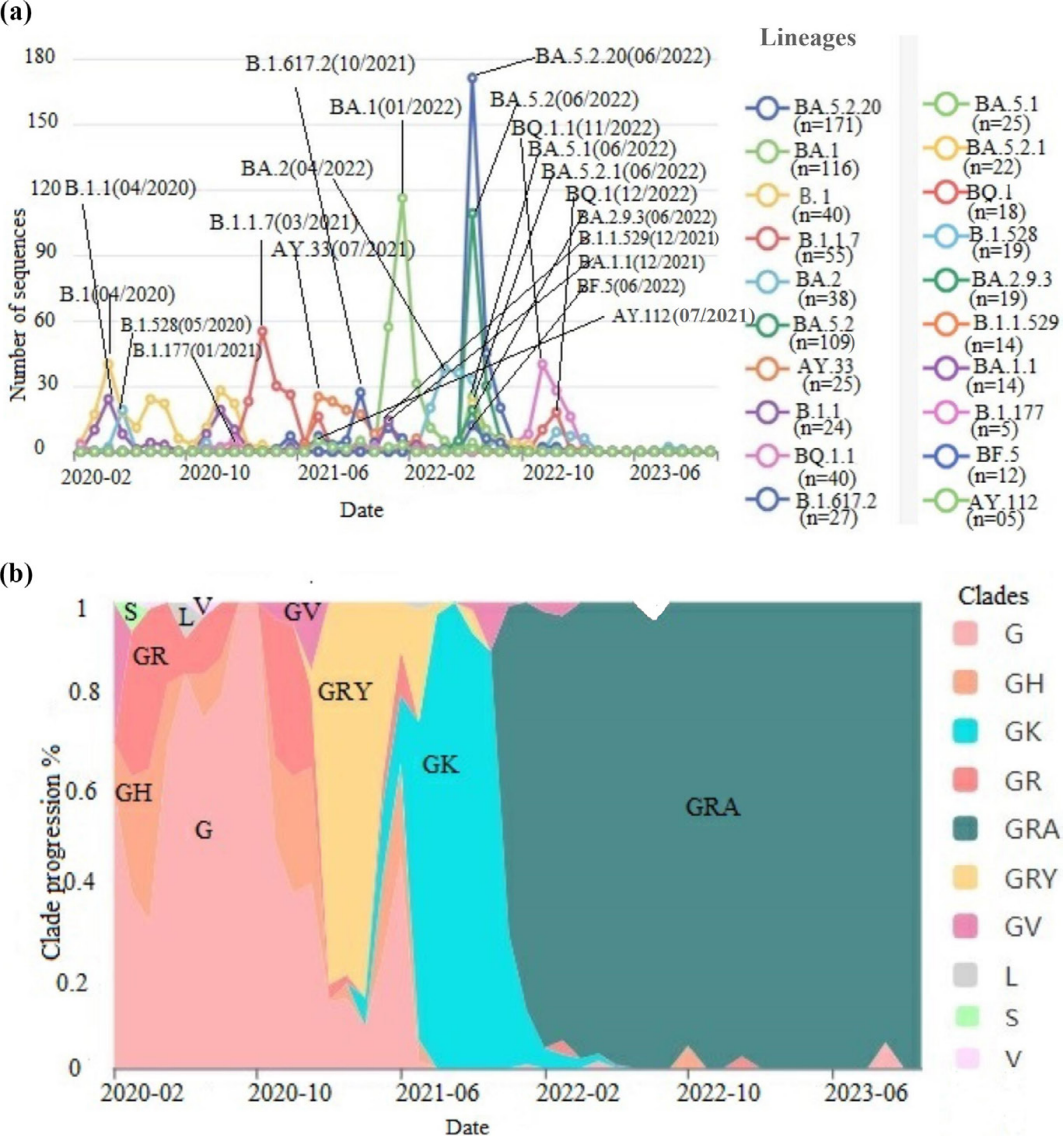
Temporal evolutionary dynamics of SARS-CoV-2 lineages and sublineages: (**a)** GISAID graph illustrating the gradual emergence of notable peaks of SARS-CoV-2 variants and lineages observed during the pandemic. (**b)** Progression of GISAID clades over the years (2020–2023).

The analysis of genomic sequences in this study has highlighted a convergent evolution of several variants, particularly the Delta and Omicron variants and their related subvariants (Table 1). In this regard, increasing diversification has been observed within the Omicron and Delta variant, and their sublineages. Over time, Omicron and its subvariants has shown increasing diversification gradually supplanted previous lineages and variants such as B.1, B.1.528, B.1.177, as well as the B.1.1.7, Delta and its subvariants (AY.*), B.1.621, B.1.525, B.1.617.1, and B.1.351 variants (Fig. 1a). The most notable peaks of Omicron and its derivatives were observed in June 2022, a period during which several co-circulating lineages entered into competition to establish their dominance (Fig. 1a and b).

Some subvariants, such as BA.5.2.20(11%), BA.1(10.1%), and B.1(9.8%), stood out due to their high frequency. Variants B.1.1.7(7.7%), BA.2(7.1%), BA.5.2(6.6%), and AY.33(5%) also exhibited notable frequencies. Other subvariants such as B.1.1, BQ.1.1, B.1.617.2, BA.5.1, BA.5.2.1, BQ.1, and B.1.528 were also noted with non-negligible frequencies (Table 3).

**Table 3.** Relative frequency of the highest genomic sequences

Pango lineage	No. of sequences	Frequency (%)
BA.5.2.20 (Omicron)	248/2274	11%
BA.1 (Omicron)	230/2274	10.1%
B.1 (lineage B.1)	223/2274	9.8%
B.1.1.7 (Alpha)	176/2274	7.7%
BA.2 (Omicron)	161/2274	7.1%
BA.5.2 (Omicron)	151/2274	6.6%
AY.33 (Delta)	112/2274	5%
B.1.1 (lineage B.1)	99/2274	4.3%
BQ.1.1 (Omicron)	96/2274	4.2%
B.1.617.2 (Delta)	75/2274	3.3%
BA.5.1 (Omicron)	44/2274	2%
BA.5.2.1 (Omicron)	39/2274	1.7%
BQ.1 (Omicron)	30/2274	1.3%
B.1.528 (lineage B.1.528)	25/2274	1.1%

### Genomic variation and mutational patterns

**Fig. 2.**
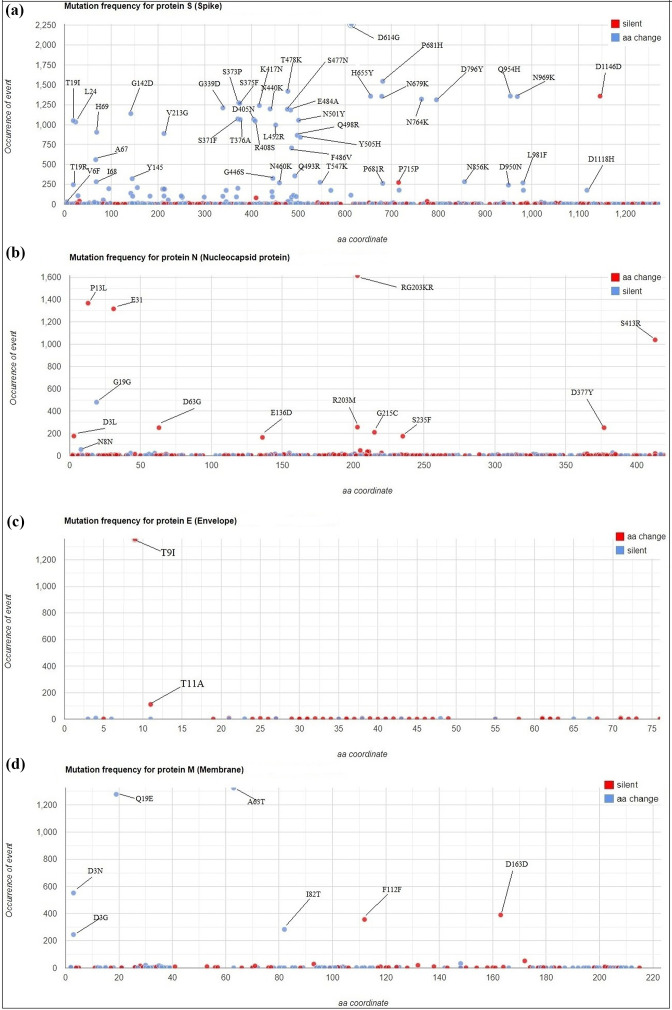
Distribution of synonymous and non-synonymous mutation frequencies of amino acids in structural proteins of the 2274 analysed genomic sequences. The coordinates on the *Y*-axis represent the different scales of mutation frequencies observed in the structural proteins of the 2274 analysed genomic sequences. The *X*-axis shows the position of each amino acid mutation along the sequences constituting the structural proteins: in the Spike protein (**a**) with ~1273 amino acids and the membrane protein (**d**) with ~222 amino acids, the blue dots represent non-synonymous mutations while the red dots indicate synonymous mutations. In the nucleocapsid protein (**b**) with ~419 amino acids and the envelope protein (**c**) with ~75 amino acids, the red dots show non-synonymous mutations while the blue dots represent synonymous mutations.

The comparison of 2274 genomic sequences with the reference genome Wuhan-Hu-1 revealed a notable genetic diversity, characterized by a substantial number of mutations, both synonymous and non-synonymous (Fig. 2).

In total, 123 530 mutations were observed in comparison with the reference genome. Among them, an important frequency of SNP was observed, representing 64.30%(79 433/123 530) of the mutations, while the proportion of silent nucleotide substitutions or variations (silent SNPs) in transcribed regions of the genome was 21.70%(26 814/123 530). Insertions and deletions (indels) were also common, with 5.4 %(6719/123 530) of deletions, 2.1 %(2583/123 530) of frameshift deletions, followed by 0.2%(229/123 530) of insertion events, and 0.6%(799/123 530) of frameshift insertions. Mutations in extragenic regions (UTR) had a prevalence of 10.7%(13 192/123 530) (File S2).

**Fig. 3.**
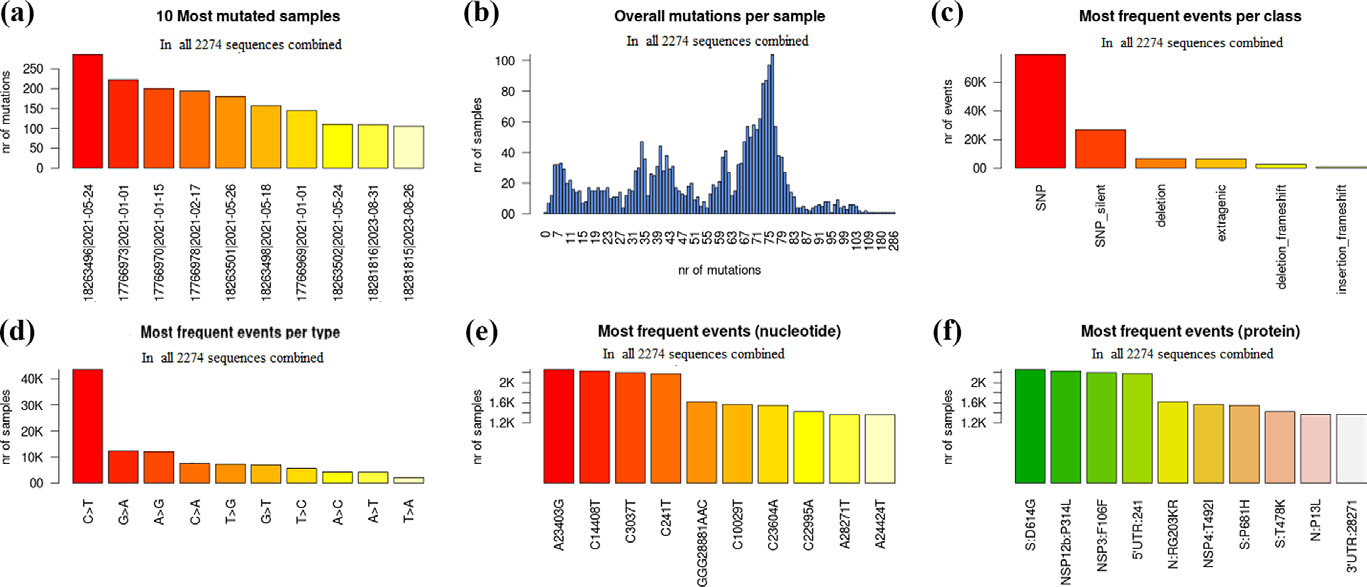
Distribution of mutational events in the 2274 analysed genomic sequences: (**a**) selection of the most mutated sequences among the 2274, (**b**) overall number of mutations per sequence, (**c**) distribution of genomic variations (SNPs and Indels), (**d**) frequency of the different types of substitutions observed, (**e**) identification of the most frequently encountered point, (**f**) analysis of the most common amino acid mutations.

Transition substitution was the most common among the 2274 sequences, with the C>T transition being the most frequent substitution event, occurring with a prevalence of 35.5%(43 862/123 530). This genomic variation was followed by G>A and A>G with frequencies of 10%(12 303/123 530) and 9.8%(12 125/123 530), respectively. The transition substitutions were followed by T>G transversion with a proportion of 5.8%(7201/123 530), G>T with a proportion of 5.6%(6997/123 530), and T>C, A>C, A>T, T>A substitutions with prevalences ranging from 1.7–4.5% (Fig. 3d).

**Fig. 4.**
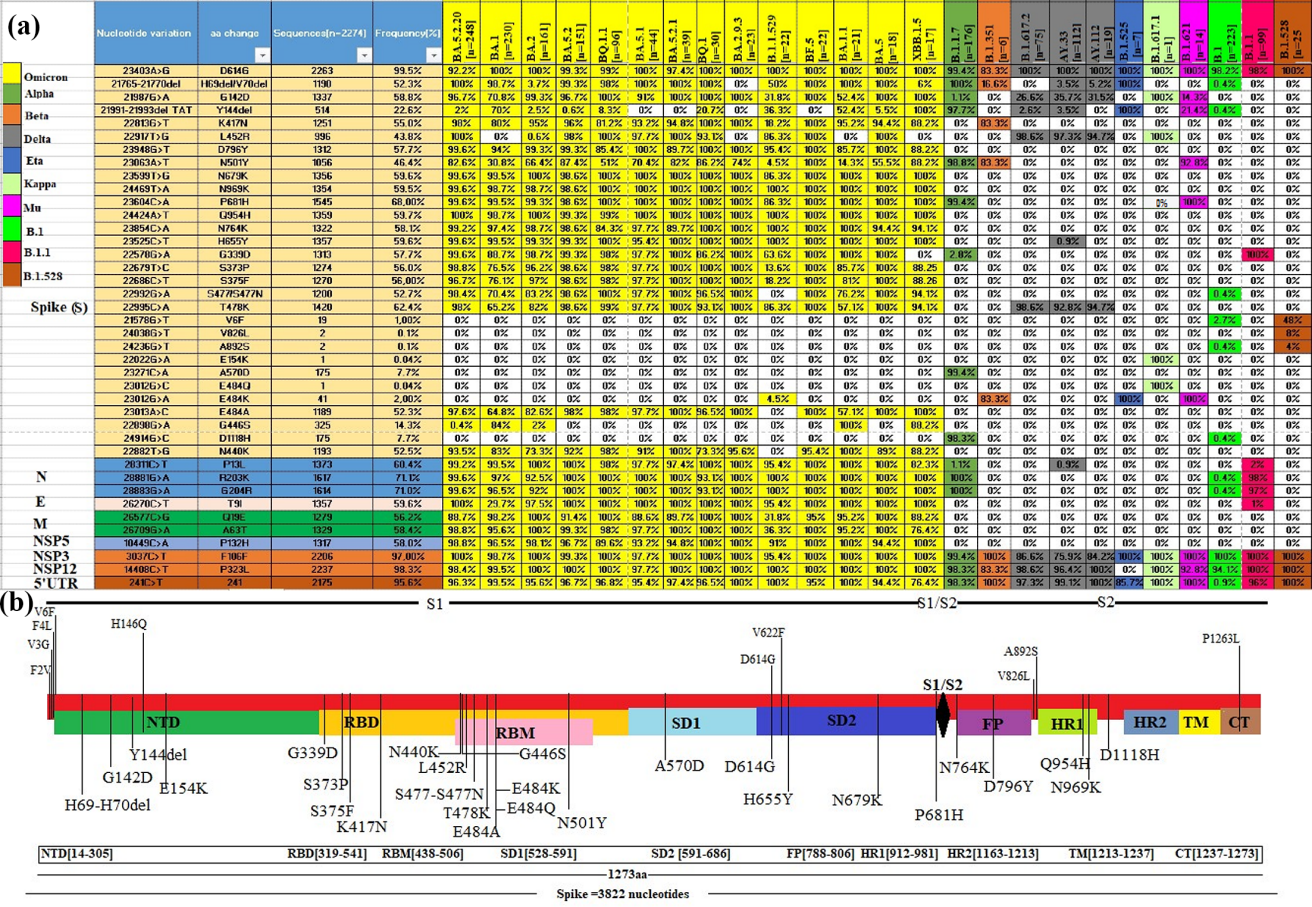
Frequencies of key amino acid mutations observed in the structural proteins (S, M, N, and E) of variants and the predominant lineages of SARS-CoV-2, as well as the localization of the mutations in the Spike glycoprotein, displayed on a schematic genomic map of SARS-CoV-2. (**a**) The amino acid mutations observed in the variants and certain predominant lineages among the 2274 genomic sequences analysed are visually represented. Each coloured box indicates the proportion of specific amino acid substitutions in these variants and lineages, while the non-coloured (white) box indicates the absence of the corresponding amino acid mutation in the respective variant or lineage. The prevalence of each mutation is assessed by determining the number of specific mutations hosted within the totality of sequences assigned to each variant or lineage. (**b**) Diagram illustrating the location of some mutations distributed in the different regions of the Spike glycoprotein. These regions include: N-terminal domain (NTD), receptor-binding domain (RBD), receptor-binding motif (RBM), fusion peptide (FP), HR1 and HR2 (heptad repeats 1 and 2), transmembrane domain (TM), and CT (cytoplasmic tail). Mutations identified in the upper part of (b) correspond to mutations in the unique B.1.528 lineage.

The genetic variations observed in the genomic sequences of SARS-CoV-2 revealed several notable events, notably the 23 403A>G (D614G) genetic variation in the Spike protein, present in 99.5%(2263/2274) of the analysed genomic sequences. This mutation was followed by the 14 408C>T variation (P314L) affecting 98.3%(2237/2274) of sequences and resulting in a change in the NSP12 protein (P323L). Silent mutations were also observed, such as 3037C>T (F106F) (97%; 2206/2274) targeting NSP3 (Fig. 3e, f). Furthermore, several other mutations affecting various protein sequences in this study were observed at notable frequencies. Among these mutations, the RG203KR mutation identified in the nucleocapsid protein (N), with a prevalence of 71%(1615/2274) and the P13L mutation with a prevalence of 60.4%(1370/2274). These mutations are followed by NSP4: T492I, with a frequency of 68.7%(1564/2274), Fig. 3f. Additionally, amino acid substitutions in the Spike glycoprotein, notably P681H (68%; 1545/2274) and T478K (62.4%; 1420/2274) were frequently observed. Mutations in non-coding regions were also identified, notably the 241C>T mutation in 5'UTR (95.6%; 2175/2274) and the 28 271A>T mutation in 3'UTR, detected in 60%(1363/2274) of the sequences analysed (Fig. 3f).

Besides the substantial mutations observed in the spike glycoprotein (Fig. 2a), notable genetic mutations have also been highlighted in the viral envelope and membrane protein (Fig. 2). Although less frequent than those observed in the Spike protein and nucleocapsid protein, these mutations have been identified at notable rates. For example, in the E protein, mutations T9I (59.6%; 1356/2274) and T11A (4.9%; 111/2274) were the most notable, while in the M protein, mutations such as Q19E (56.3%; 1280/2274), A63T (58.1%; 1323/2274), D3N (24.2%; 550/2274), and D3G (10.7%; 243/2274) were also observed with notable frequencies (Fig. 2c, d).

### Characteristics of the most important mutations in the predominant SARS-CoV-2 lineages isolated in Morocco

Among the numerous variants identified in this study, Omicron and its subvariants harboured the highest rate of mutations. These mutations are localized in different domains of the genome, among which those requiring particular attention are found in the Spike glycoprotein. In the N-terminal domain (NTD) of the S1 subunit, are observed mutations such as T19I, L24del, P25del, P26del, A27S, Q52H, A67V, H69del/V70del, T95I, G142D, Y144del, H146Q, E180V, Q183E, N211del, L212I, V213E, ins214EPE, G252V, and D253G. Within the RBD/RBM domain (S1 subunit), some sequences exhibited mutations such as G339D/H, R346T, S371F, T376A, D405N, R408S, K417N, N440K, K444T, V445P, G446S, L452R, F456L, N460K, S477/S477N, T478K, E484A, F486P/S/V, F490S, Q493R, Q498R, N501Y, and Y505H were identified. Mutations are also present in the SD1 and SD2 domains (S1 region) outside RBD/RBM domain such as P521S, D614G, A626V, H655Y, N679K, P681H, and S704L while the S2 subunit harbours mutations such as N764K, D796Y, N969K, and Q954H (Figs3a  4a and File S3).

As for the Delta variant and its subvariants (AY.*), they harboured notable mutations such as G142D, T478K, and L452R (Fig. 4a). Other notable mutations were also identified, including T19R, T29A, F157del, R158del, E156G, T250I, T299I, Q613H, P681R, and D950N. The Alpha and Beta variants carried mutations such as H69del/V70del, Y144del, N501Y, A570D, D614G, P681H, and D1118H for the Alpha variant, while the Beta variant displayed mutations like D614G, N501Y, L18F, D80A, G142D, L242del/L244del, K417N, and E484K (Figs3a  4a and File S2).

However, the B.1.525, B.1.617.1, and B.1.621 variants stood out due to mutations such as A67V, H69del/V70del, Y144del, E484K, and D614G, hosted mutations in the spike protein of the Eta variant, and E154K, E484Q, G142D, L452R, D614G, P681R, Q1071H, and T95I for Kappa. The Mu variant was characterized by mutations such as T95I, A262S, D614G, D950N, E484K, N501Y, P681H, R346K, and Y144del (Figs3a  4a).

### Phylogenetic analysis of the 2274 genomic sequences

**Fig. 5.**
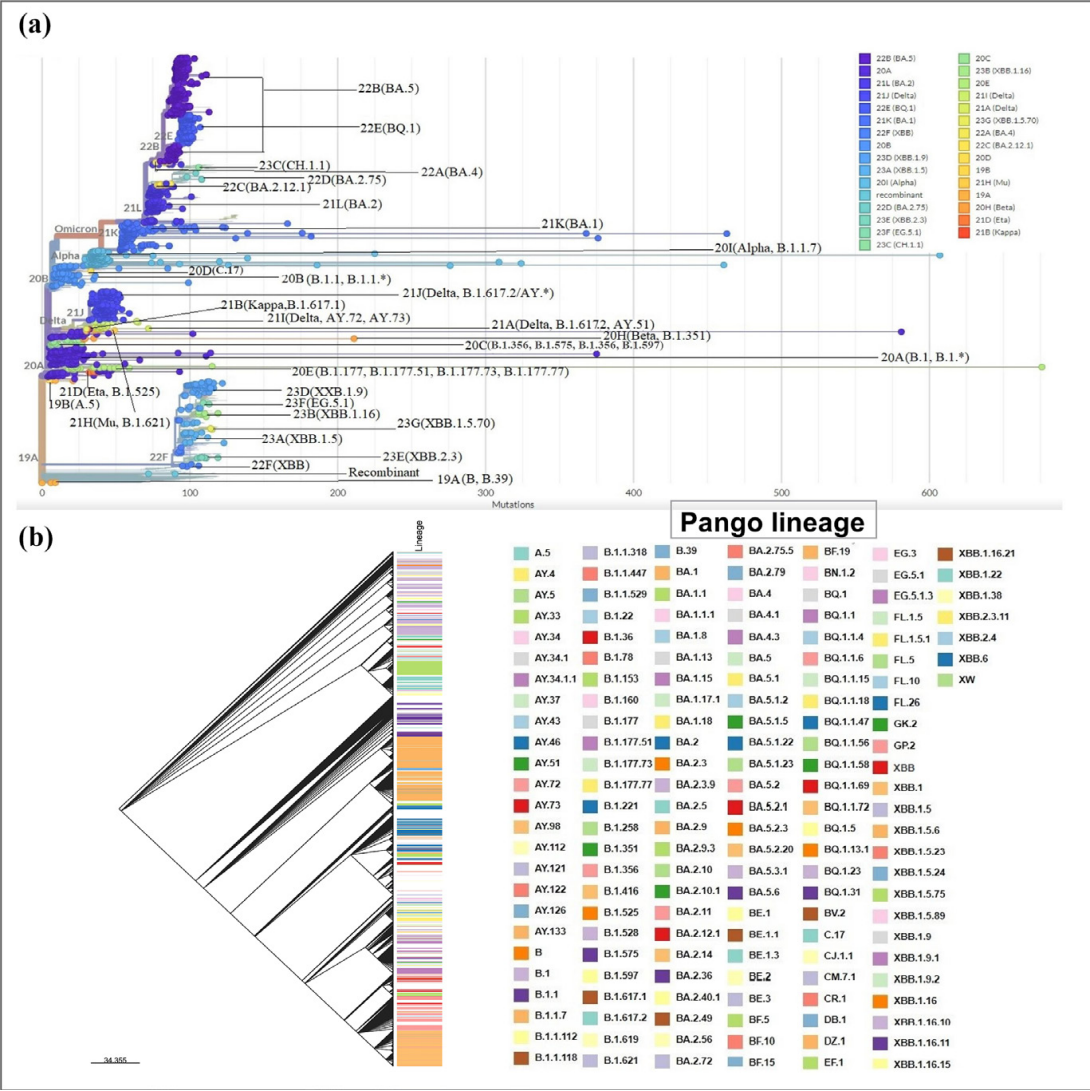
Phylogenetic tree generated from 2274 genomic sequences of Moroccan isolates. (**a)** Maximum-likelihood cladistic phylogenetic tree generated by Nextclade. (**b)**. Phylogenetic tree of the 2274 genomic sequences and attribution of different Pango lineages performed using Microreact.

To analyse the phylogenetic characteristics of the 2274 SARS-CoV-2 sequences, a phylogenetic tree was generated using the maximum-likelihood method, aligned with the reference genomic sequence. The analysis revealed the presence of 157 PANGO lineages grouped within thirty distinct clades. Among the 157 identified lineages, one (recombinant XW) has not been assigned to a specific clade (Fig. 5a and b, Table 4).

**Table 4.** Distribution of the different clades and related phylogenetic lineages

Clade_display	Pango lineage
19A	B, B.39
19B	A.5
20A	B.1, B.1.22, B.1.36, B.1.78, B.1.153, B.1.160, B.1.221, B.1.258, B.1.416, B.1.528, B.1.619
20B	B.1.1, B.1.1.112, B.1.1.118, B.1.1.318, B.1.1.447
20C	B.1.356, B.1.575, B.1.597
20D	C.17
20E	B.1.177, B.1.177.51, B.1.177.73, B.1.177.77
20 H (Beta)	B.1.351
20I (Alpha)	B.1.1.7
21A (Delta)	B.1.617.2(13 sequences), AY.51
21B (Kappa)	B.1.617.1
21D (Eta)	B.1.525
21 H (Mu)	B.1.621
21I (Delta)	B.1.617.2(2 sequences), AY.37, AY.72, AY.73, AY.133
21J (Delta)	B.1.617.2(60 sequences), AY.4, AY.5, AY.33, AY.34, AY.34.1, AY.34.1.1, AY.43, AY.46, AY.98, AY.112, AY.121, AY.122, AY.126
21K (BA.1)	BA.1, BA.1.1, BA.1.1.1, BA.1.8, BA.1.13, BA.1.15, BA.1.17.1, BA.1.18
21L (BA.2)	BA.2, BA.2.3, B.1.1.529, BA.2.3.9, BA.2.5, BA.2.9, BA.2.9.3, BA.2.10, BA.2.10.1, BA.2.11, BA.2.14, BA.2.36, BA.2.40.1, BA.2.49, BA.2.56, BA.2.72, BA.2.79, CM.7.1
22A (BA.4)	BA.4, BA.4.1, BA.4.3
22B (BA.5)	BA.5, BA.5.1, BA.5.1.2, BA.5.1.5, BA.5.1.22, BA.5.1.23, BA.5.2, BA.5.2.1, BA.5.2.3, BA.5.2.20, BA.5.3.1, BA.5.6, BE.1, BE.1.1, BE.1.3, BE.2, BE.3, BF.5, BF.10, BF.15, BF.19, BV.2, CR.1, DB.1, DZ.1
22C (BA.2.12.1)	BA.2.12.1
22D (BA.2.75)	BA.2.75.5, BN.1.2, CJ.1.1
22E (BQ.1)	BQ.1, BQ.1.1, BQ.1.1.4, BQ.1.1.6, BQ.1.1.15, BQ.1.1.18, BQ.1.1.47, BQ.1.1.56, BQ.1.1.58, BQ.1.1.69, BQ.1.1.72, BQ.1.5, BQ.1.13.1, BQ.1.23, BQ.1.31, EF.1
22F (XBB)	XBB, XBB.1, XBB.1.22, XBB.1.38, XBB.2.4, XBB.6
23A (XBB.1.5)	XBB.1.5, XBB.1.5.6, XBB.1.5.23, XBB.1.5.24, XBB.1.5.89, XBB.1.5.75
23B (XBB.1.16)	XBB.1.16, XBB.1.16.10, XBB.1.16.11, XBB.1.16.15, XBB.1.16.21
23C (CH.1.1)	GP.2
23D (XBB.1.9)	XBB.1.9, EG.3, FL.1.5, FL.1.5.1, FL.5, FL.10, FL.26, XBB.1.9.1, XBB.1.9.2
23E (XBB.2.3)	XBB.2.3.11
23F (EG.5.1)	EG.5.1, EG.5.1.3
23G (XBB.1.5.70)	GK.2
Unassigned clade	Recombinant XW

The phylogenetic analysis revealed a gradual diversification of the virus over time, characterized by the emergence of one of the earliest mutations, D614G, in the Spike glycoprotein. This mutation greatly enhanced the virus’s transmission capacity, marking the onset of high-frequency spread of its first evolutionary clone. Initially (in 2020) seven distinct clades were identified with lineage B (clade 19A), considered as the parental lineage of the reference strain Wuhan-Hu-1, followed by subsequent lineages like B.1 and B.1.528 from clade 20A; B.1.1/B.1.1.* from clade 20B; B.1, B.1.356, B.1.575 from clade 20C; C.17 (clade 20D), and B.1.177, B.1.177.73, B.1.177.77 from clade 20E (Fig. 5a and b). Lineage C.17 (clade 20D), partially aliased to B.1.1.1.17, is similar to the lineage that emerged in Egypt, suggesting an introduction event from Egypt. Substantial spread was observed from clades 20A and 20B through community transmission. Notably, lineage B.1.528 formed monophyletic clusters nationwide. The lineages from the aforementioned clades became less frequent in the last quarter of 2020. Over time, numerous VOCs have been identified. One of the earliest VOCs identified in Morocco was the Alpha variant (clade 20I), isolated in the first quarter of 2021, accounting for 7.7% of all analysed genomic sequences (Table 2). Subsequently, Morocco has witnessed the emergence of other substantial variants, as shown in Fig. 5a and b.

**Fig. 6.**
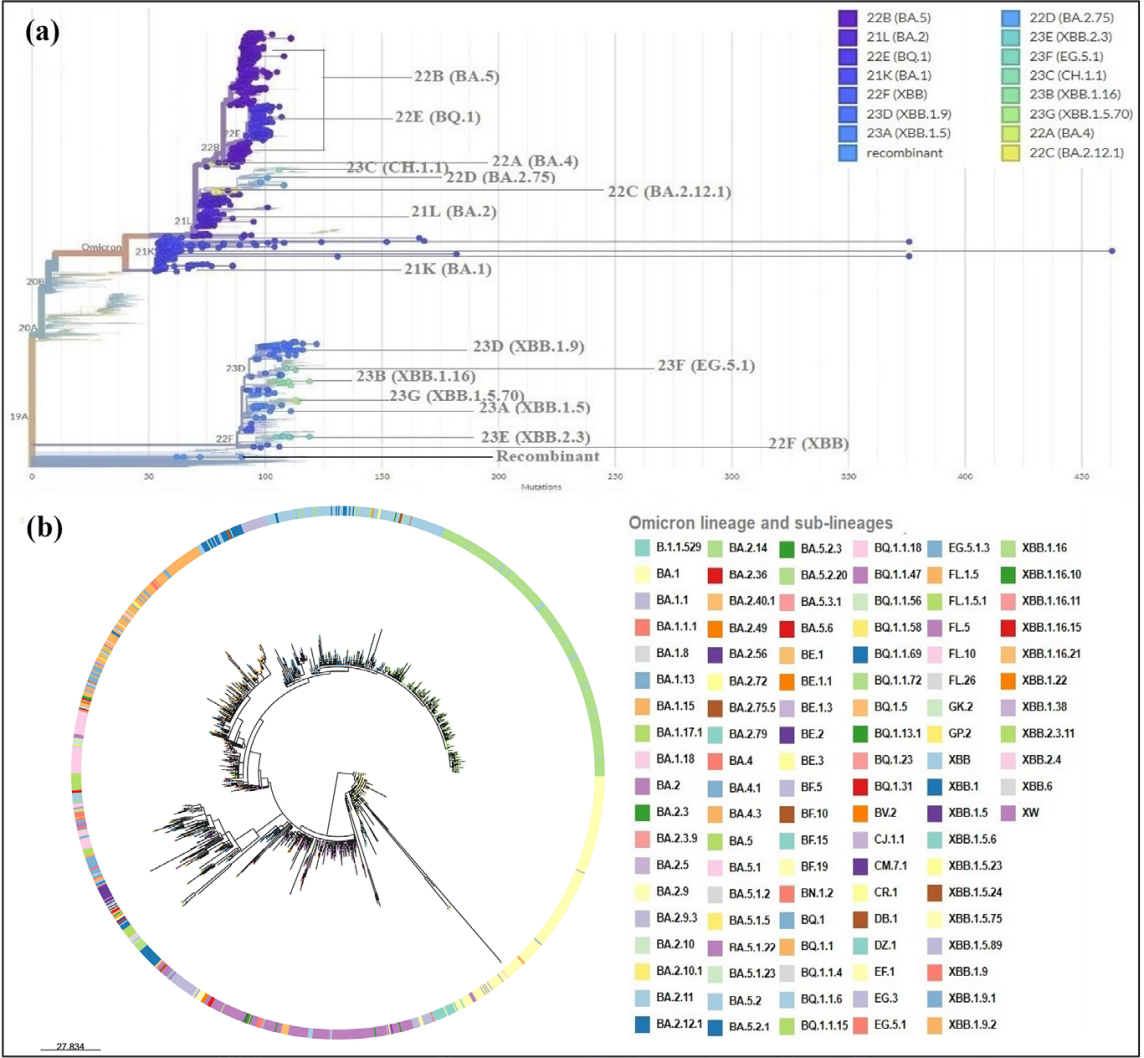
Estimated maximum-likelihood phylogeny of convergent evolution of Omicron strain and its progeny isolated in Morocco. (**a**) Phylogenetic tree of Omicron isolates generated by Nextclade, illustrating their classification into different clades based on their evolutionary relationships. (**b**) Phylogenetic tree of the Omicron lineage and its derivatives generated by Microreact. The phylogenetic tree generated from the 1353 sequences attributed to Omicron and its subvariants show how these variants are linked by common ancestors, illustrating their genetic diversity and evolutionary relationships.

The Beta variant from clade 20H had a relatively low prevalence of 0.26%, while the Eta variant from clade 21D had a proportion of 0.31 % of all genomic sequences. Additionally, the Kappa variant (clade 21B) resulted from an introduction event in Morocco with a single identified sequence (0.04%), and was closely related to the B.1.617.1 sequence from India. On the other hand, the Mu variant from clade 21H accounted for 0.62% of the analysed sequences and was closely related to B.1.621 sequences from Colombia. As for the Delta variant and its subvariants (AY.*), their prevalence was 11.30%. Furthermore, the phylogenetic analysis of the Delta variant and its subvariants revealed varied distribution in three distinct clades, namely 21A, 21I, and 21J in comparison with the original strain (Fig. 5a), highlighting several phylogenetically distinct clusters. Towards the end of the year 2021, Omicron and its subvariants (Fig. 5a) emerged spectacularly, representing 59.5% of all genomic sequences analysed. This emergence has been marked by the presence of 15 distinct clades (21K, 21L, 22A, 22B, 22C, 22D, 22E, 22F, 23A, 23B, 23C, 23D, 23E, 23F, and 23G) constituting several phylogenetically distinct groups, suggesting multiple noteworthy genetic divergences and different introduction events (Fig. 6a). In the following lines, the emergence of Omicron and its subvariants will be analysed in more detail.

### Phylogenetic analysis of genomic diversity of the Omicron variant

Among the 2274 genomic sequences analysed, 1353 sequences were attributed to the Omicron variant and its subvariants (Table 1). A total of 96 450 mutation events were identified, with the majority being SNPs representing 66.2%(63 899/96 450) of all events, followed by silent SNPs with a prevalence of 21.5%(20 808/96 450). SNPs involving a stop codon represented only 0.1%(10/96 450) of all events. Transition-type substitutions were the most frequent. The C>T transition was the most frequent event with a prevalence of 34.9%(33 673/96 450). Subsequently, G>A and A>G transitions were observed with frequencies of 11.5%(11 114/96 450) and 9.7%(9386/96 450), respectively. Among transversion substitutions, the most notable event was T>G, accounting for 6.8%(6621/96 450) of all mutations.

The phylogenetic analysis of Omicron evolution and its subvariants, as illustrated in Fig. 6a and b, revealed a total of 106 Pango lineages grouped into 15 distinct Nextstrain clades, along with one recombinant (XW) lineage with an unassigned clade. Clades 21K (BA.1), 21L (BA.2), 22A (BA.4), 22B (BA.5), 22C (BA.2.12.1), 22D (BA.2.75), 22E (BQ.1), and 23C (CH.1.1) are the progeny of clade 20B, as illustrated in Fig. 6a. In particular, clades 21K, 21L, and 22B, phylogenetically related to BA.1, BA.2, and BA.5 lineages, respectively, showed a remarkable evolution in Morocco throughout the year, with a notable number of infection cases recorded. In addition, Omicron variant BQ.1 and one of its sublineages, BQ.1.1, both descendants of BA.5, were among the dominant subvariants emerging towards November 2022. Clade 22F, phylogenetically related to recombinant XBB, commonly known as «gryphon », is one of Omicron’s subvariants with several progenies such as XBB.1 (clade 22F) «hippogryph», 23A (XBB.1.5) « kraken», and 23B (XBB.1.16) «Arcturus» etc. These sublineages are, respectively, considered grandparent, parent, and sublineage, respectively. Furthermore, clades such as 23D (XBB.1.9), hosting various subvariants including XBB.1.9.2, as well as clade 23F, hosting variant EG.5.1 named «Eris» and its sublineages (Fig. 6a). Conversely, subvariants within the clades 22F (XBB), 23A (XBB.1.5), 23B (XBB.1.16), 23D (XBB.1.9), 23E (XBB.2.3), 23F (EG.5.1), 23G (XBB.1.5.70) as well as the recombinant XW have circulated sporadically in Morocco between the last quarter of 2022 and 2023.

### Genomic analysis of the lineage B.1.528

The analysis of the 25 genomic sequences of the B.1.528 lineage revealed an important proportion of genetic polymorphism in comparison with the reference sequence. A total of 231 mutation events were identified, with SNPs representing 44.6%(103/231) of all events. Silent SNPs were observed with a frequency of 27.2%(63/231), while deletions and insertions had a prevalence of 3.9%(9/231), respectively. In addition, mutations in extragenic regions (UTR region) had a prevalence of 20.3%(47/231). Transition-type substitutions were the most common event. The C>T substitution was the most frequent event with 49.3%(114/231). This genomic variation was followed by T>A transversion events with a frequency of 12.1%(28/231). The notable mutations detected in the B.1.528 lineage are listed in Table 5.

**Table 5.** Notable mutations observed in the lineage B.1.528

Genomic region	Nucleotide variation	Amino acid change	No. of sequences	Frequency (%)	Genetic variations	Mutation type
**Spike protein**	A23403G	S: D614G	25	100%	SNP	Non-synonymous
**Spike protein**	G24038T	S: V826L	2	8%	SNP	Non-synonymous
**Spike protein**	G21578T	S: V6F	5	20%	SNP	Non-synonymous
**Spike protein**	C25350T	S: P1263L	2	8%	SNP	Non-synonymous
**Spike protein**	G23426T	S: V622F	1	4%	SNP	Non-synonymous
**Spike protein**	T21572C	S: F4L	1	4%	SNP	Non-synonymous
**Spike protein**	C22000A	S: H146Q	1	4%	SNP	Non-synonymous
**Spike protein**	T21566G	S: F2V	1	4%	SNP	Non-synonymous
**Spike protein**	T21570G	S: V3G	1	4%	SNP	Non-synonymous
**Spike protein**	G24236T	S: A892S	1	4%	SNP	Non-synonymous
**Nucleocapsid protein**	C29421T	N: P383L	2	8%	SNP	Non-synonymous
**NSP2**	G2458T	ORF1ab/NSP2: M551I	2	8%	SNP	Non-synonymous
**NSP3**	C3037T	ORF1ab /NSP3: F106F	25	100%	SNP	Synonymous
**NSP6**	C11139T	ORF1ab /NSP6: A56V	1	4%	SNP	Non-synonymous
**NSP6**	G11306A	ORF1ab /NSP6: D112N	1	4%	SNP	Non-synonymous
**NSP6**	T11309A	ORF1ab /NSP6: C113S	1	4%	SNP	Non-synonymous
**NSP12**	C14408T	ORF1ab /NSP12: P323L	25	100%	SNP	Non-synonymous
**NSP14**	C18508T	ORF1ab /NSP14: L157F	25	100%	SNP	Non-synonymous
**NSP14**	T19420A	ORF1ab /NSP14: S461T	1	4%	SNP	Non-synonymous
**NSP14**	T19420C	ORF1ab /NSP14: S461P	1	4%	SNP	Non-synonymous
**NSP14**	C18568T	ORF1ab /NSP14: L177F	1	4%	SNP	Non-synonymous
**NSP15**	G19656T	ORF1ab /NSP15: K12N	1	4%	SNP	Non-synonymous
**5’UTR**	C241T	5’UTR: 241(NA)	25	100%	Extragenic	Non-coding

SNP=Single Nucleotide Polymorphism, NSP=Non-Structural Protein.

**Fig. 7.**
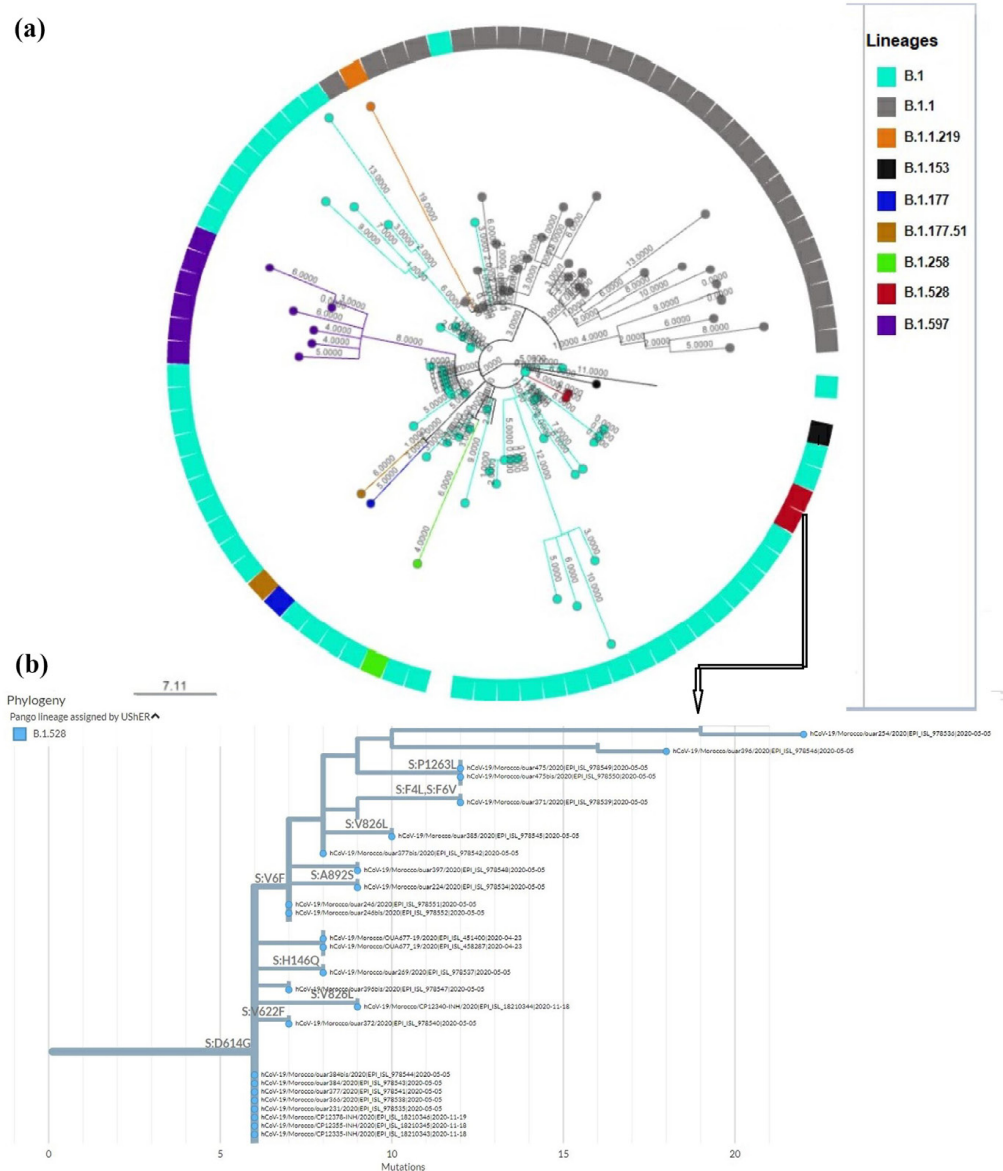
Maximum-likelihood phylogenetic tree analysis of 89 genomic sequences of SARS-CoV-2 isolated and sequenced in Morocco in 2020. (**a**) Phylogenetic tree of lineages isolated in 2020. (**b**) The lineage « B.1.528 » with notable amino acid substitution observed in the 25 isolates.

The lineage « B.1.528 » constituted a monophyletic cluster within the Moroccan population (Fig. 7a), with a proportion of 1.1%(25/2274). Twenty-four sequences of the B.1.528 lineage were detected in Ouarzazate and one sequence in Rabat. The analysis of the phylogenetic tree based on 89 sequences isolated in 2020 reveals that lineage B.1.528 evolved from the ancestral lineage, displaying several additional distinct mutations in addition to the initial D614G mutation, from which many other lineages have emerged (Fig. 7a and b). Moreover, the emergence of the B.1.528 lineage has sparked increasing interest due to the identification of specific mutations in its genomic sequence. These include the D614G mutation located in the S1 domain, followed by mutations such as F2V, V3G, F4L, and V6F in the NTD domain (S1 subunit). Additionally, mutations V826L and A892S have been observed in the S2 domain, along with the amino acid substitution of P1263L in the C-terminal region (Fig. 7b). Fig. 7b provides a detailed insight into the mutations present in the 25 sequences of B.1.528, illustrating the genetic diversity and evolutionary dynamics of this lineage. These mutations observed through the tree, provide substantial information on the evolution and differentiation of key mutations within the lineage B.1.528 over time.

## Discussion

This study stands out as one of the first to investigate the complete genomic divergence of 2274 sequences of severe acute respiratory syndrome coronavirus 2 in Morocco over the 3 years following the COVID-19 pandemic. Furthermore, this study highlights the impact of SARS-CoV-2 evolution on public health by identifying several distinct mutational signatures that characterize notable variants, thus providing a solid basis for future research.

During the COVID-19 pandemic, the year 2020 witnessed a turning point in the evolution of SARS-CoV-2 with the emergence of the D614G mutation [17]. Some mutations in the genome of SARS-CoV-2 have been recognized as determining mutations in variations in transmissibility (more or less contagious), antigenicity variation (immune evasion capacity and/or risk of reinfections), as well as variations in pathogenicity (virulence variation with more or less severe events) [17,19]. Mutations in the RBD/RBM region could impact how the virus interacts with the ACE2 receptor, potentially influencing the virus’s transmissibility and could assist the virus to escape immune recognition. Moreover, mutations near the furin cleavage site (S1/S2) could accelerate the Spike protein cleavage, enhancing virus infectivity (Fig. 4b) [17,19].

In Morocco in 2020, dominant lineages such as, B.1, B.1.528, B.1.177, and B.1.1 with the D614G mutation were prevalent, mainly in clade G. All subsequent SARS-CoV-2 lineages/variants in Morocco are derived from lineage B.1 (clade 20A). These lineages emerged approximately at the same time, indicating multiple introduction events in Morocco, except for the lineage « B.1.528 », which is closely related to clade 20A. This lineage formed a distinct cluster nationally and carried notable mutations in Spike glycoprotein, including D614G, V826L, and A892S (Fig. 4b and Table 5) [20]. These amino acid substitutions, located outside the predicted immunological epitope region, could affect transmissibility and immune evasion [20]. Furthermore, this study highlighted the P1263L mutation, located in the C-terminal region, within B.1.528 sequences. This mutation has also been reported in certain lineages in Europe and Oceania [21]. Interestingly, previous studies have suggested that the P1263L mutation, could potentially alter the structure and function of the SARS-CoV-2 Spike protein, leading to decreased infectivity of the virus [21,22]. It should also be noted that non-synonymous mutations such as F2V, V3G, F4L, V6F, and H146Q in the B.1.528 sequences are located within the region of the predicted epitopes [23]. Previous studies have reported that these mutations could contribute to increased transmissibility and pathogenicity of SARS-CoV-2 [23]. Moreover, their location within epitopes could potentially confer to the virus the ability to escape the immune system [21,22]. Consequently, all these key mutations in the spike glycoprotein, particularly those associated with the D614G mutation present in the sequences of lineage B.1.528 (Fig. 7b and Table 5), played a crucial role in the emergence of this lineage within clade 20A. These mutations distinguish it from other lineages within the same clade, as well as from lineages of ancestral clades such as 19A, 19B, 20B, and 20C, where the dominant mutation in the Spike protein was sometimes only the D614G mutation (Table 4).

In January 2021, the Alpha variant (B.1.1.7), previously designated as VOC 202012/01, and commonly known as GRY clade (formerly GR/501Y.V1), was identified in Sidi Lahcen, Morocco. Known for its increased transmissibility, Alpha variant led to strengthened health measures in Morocco. The N501Y mutation in the Spike protein, found in the Alpha, Beta, Mu, and Omicron variants (Fig. 4a), is known to enhance virus transmissibility [17,24]. In addition, it has been reported that the deletions such as H69del/V70del and Y144del in the NTD region would be involved to immune evasion and increased virulence of SARS-CoV-2 [18,24]. The P681H substitution in the S1/S2 cleavage loop, observed in several variants, would favour the processivity of Spike protein cleavage [17,19, 25]. Moreover, the coexistence of the N501Y and P681H mutations would increase transmission and disease severity [17,19, 25]. However, it is interesting to note that the introduction of the Alpha variant (B.1.1.7) marked a major change in the evolutionary dynamics of SARS-CoV-2 in Morocco. Furthermore, our observations corroborate the findings of a previous study, which reported that at the beginning of 2021, the Alpha variant was dominant in Tunisia, Algeria, and Morocco, while the Eta variant predominated in Egypt, Libya, and Mauritania [26]. In contrast, while Hamzaoui and colleagues observed a frequency of less than 1% for the Alpha variant and an absence of sequences attributed to the Beta variant, our study showed a prevalence of 7.74% for the Alpha variant and 0.31% for the Beta variant [26].

In May 2021, the Beta variant (B.1.351) of SARS-CoV-2 has been detected in Casablanca, Morocco. This variant is characterized by key mutations in the RBD/RBM (N501Y, E484K, K417N) and NTD (L242del/L244del) regions. Previous studies have suggested that these mutations may contribute to SARS-CoV-2′s immune evasion and increased risk of reinfections [19,24, 27]. However, despite concerns about B.1.351 in some countries, the impact of B.1.351 in Morocco has been relatively minor [28,29]. Morocco later detected the Eta, Kappa, and Mu variants. Eta, formerly known as G/484K.V3, with the first case identified in Morocco in March 2021, carries the E484K mutation known for its role to circumvent host immune responses [17,19, 24]. The Mu variant (B.1.621), previous VOI, was identified in Morocco on 22 May 2021. This variant (B.1.621) contains several notable mutations, including T95I, A262S, D614G, D950N, E484K, N501Y, P681H, R346K, and Y144del, which are likely to increase immune evasion and virus transmissibility [30,31]. In general, the Beta, Eta, Kappa, and Mu variants did not have a major impact in Morocco, possibly due to effective health measures or competition with more widespread variants such as Alpha, Delta, and Omicron.

In the last quarter of 2020, the epidemiological dynamics of COVID-19 have evolved substantially. India, in particular, has faced an outbreak of several SARS-CoV-2 variants that have spread to affect many countries. Among these variants, B.1.617.1 and B.1.617.2 emerged, both sharing key mutations such as G142D, L452R, and P681R in the spike glycoprotein, along with other distinct mutations likely to increase virus transmission and promote immune evasion [17,19, 32,35]. It is important to note that the Kappa and Delta variants have been labelled under the generic name of «double mutants», due to the presence of key mutations L452R and E484Q (Fig. 4a), which had never been observed together before the emergence of these variants [17,19, 32,35]. However, the epidemiological situation in Morocco in 2021 was marked by the emergence of B.1.617.2 variant and its subvariants (B.1.617.2/AY.*), which are phylogenetically related to clades 21A, 21I, and 21J. The first strain of the Delta variant (B.1.617.2) was isolated on 22 April 2021, in Casablanca. Following their introduction, they progressively became the predominant variant, supplanting other variants. Between June and September 2021, B.1.617.2 underwent rapid evolution, giving rise to a major evolutionary node with the emergence of subvariants such as AY.33, AY.112, and AY.122, which became dominant nationwide. Compared to the double mutant B.1.617.1, the B.1.617.2 variant and its subvariants harboured mutations in the NTD domain such as T19R, T29A, G142D, F157del, E156G, R158del, T250I, T299I, and in the RBD domain with mutations L452R and T478K. The SD2 region also revealed mutations such as Q613H, D614G, D950N, as well as an amino acid substitution around the furin cleavage site, P681R (mutation shared by Delta and Kappa). All these mutations differentiated them from other variants by their ability to enhance their transmissibility and their capacity to escape the immune system. Although the Delta variant is devoid of the E484Q mutation, analogous to the immune escape mutation « E484K » found in other variants such as B.1.351 and B.1.525, respectively. The T478K mutation hosted in the Spike glycoprotein could accelerate the replication and transmissibility of the pathogen [17,36]. Analogously to the D614G mutation, it is hypothesized that the Q613H mutation may enhance the virulence of SARS-CoV-2 by facilitating the cleavage of the spike protein, thereby enhancing fusion between the viral envelope and the cell membrane [37]. Interestingly, among the mutations of the aforementioned Delta subvariants, mutations such as T29A, T250I, T299I, and Q613H were specific to subvariants AY.33, AY.112, and AY.122. These mutations may confer to these subvariants a selective advantage by enhancing their transmission capacity and their ability to escape the immune system [38]. Moreover, previous studies have reported that the F157del and R158del deletions, as well as the L452R and T478K mutations (Fig. 4a), would confer to Delta and its subvariants the ability to escape neutralizing antibodies [19,36]. Consequently, all these characteristics make the Delta variant and its subvariants (AY.*) one of the most virulent variants of SARS-CoV-2 [39]. Furthermore, it should be noted that during the emergence of the Delta variant and its subvariants, the AY.33 and B.1.617.2 lineages predominated in Morocco (Fig. 1a and Table 3), while in Egypt and Algeria, the B.1.617.2 variant was dominant. In contrast, the AY.4, AY.122, and AY.34.1 subvariants were the most dominant in Libya, Tunisia, and Mauritania, respectively [26]. These observations highlight the distinct genetic diversity, reflecting the broader global trends in the evolution of the Delta variant and its subvariants [26].

The epidemiological evolution observed in Morocco during the last quarter of 2021 was marked by a transitional period with the emergence of the Omicron variant and its derivatives designated under the GRA nomenclature in GISAID (formerly GR/484A). As of 2022, 82.3% of genomic sequences were assigned to omicron and its subvariants (Table 1). The introduction of the Omicron variant and its subvariants in Morocco resulted in a major epidemiological shift, relegating the previously dominant Delta variant to a marginal position. Among the Omicron strains that have led to substantial infection in Morocco, we note BA.1 and BA.1.1 (displays clade 21K), as well as BA.2 and B.1.1.529 (clade 21L), identified in Morocco in December 2021. However, in June 2022, Morocco rapidly observed a surge in infection cases, with the emergence and concurrent coexistence of subvariants from clade 22B such as BA.5.2.20, BA.5, BA.5.1, BA.5.2.1, and BF.5 (subvariants emerged from Botswana, Hong Kong, and South Africa), succeeding the lineages BA.1 and BA.2, respectively (Fig. 1a and Table 4). Several derivatives of BA.1 subvariant (clade 21K) were also identified, notably the sublineage BA.1.1 (BA.1+R346K), distinguished from BA.1 by the additional presence of mutation R346K. In Morocco, alongside lineage BA.1, lineage BA.2 and its sublineages emerged, identified in February 2022, with a peak reached on April 2022. Lineage BA.1 harbours certain mutations, such as A67V, H69del/V70del, T95I, Y144del, L212I, N211del, ins214EPE, S371L, G446S, G496S, T547K, N856K, L981F, distinguishing it distinctly from lineage BA.2. Conversely, lineage BA.2 is distinguished from BA.1 by specific mutations such as T19I, L24del, P25del/P26del, A27S, V213G, S371F, T376A, D405N, R408S. Our analysis results corroborate the conclusions reported by Balupuri and colleagues in 2023 [40].

Interestingly, variants BA.1, BA.2, BA.4, and BA.5 share several mutation similarities. The mutations L452R and F486V are specific to BA.4 and BA.5, while H69del/V70del are specific to BA.1, BA.4, and BA.5. However, the Q493R mutation is specific to BA.1 and BA.2 [41,42]. Moreover, subvariants BA.4 and BA.5 also display mutation similarities in their Spike protein with previous VOCs. These mutations include the N501Y mutation hosted by the Alpha, Beta, and Mu variants; H69del/V70del hosted by the Alpha variant; K417N observed in sequences of Beta variant; E484K present in sequences of the Beta and Eta variants, while L452R and T478K are observed in sequences of the Delta variant (Fig. 4a). Unlike the Beta variant, which features the E484K mutation, subvariants BA.4 and BA.5 harbour the E484A mutation (Fig. 4a). The mutation profiles described in this study align with findings reported in previous studies [27,41,45]. Over time, the BA.2 variant has gradually emerged as the predominant subvariant, relegating the BA.1, BA.1.1, and B.1.1.529 variants to a marginal position (Fig. 1a). Furthermore, a notable descendant of the BA.2 lineage, the subvariant BA.2.12.1 (BA.2+L452Q+S704L), is characterized by an additional mutation, L452Q and S704L, absent in BA.2. Nevertheless, most lineages such as BA.2 and its sublineages, XBB and its derivatives, the Beta, Kappa, Mu, B.1, Delta variants and their derivatives were mostly devoid of the H69del/V70del mutation, unlike some Omicron subvariants, the Alpha and Eta variant (Fig. 4a). These observations correlate with previous research findings [41,46, 47]. In addition to the previously mentioned subvariants of Omicron, BA.5 and BA.4 are among those being closely monitored. In the same perspective, the BA.5.2.20 subvariant, a progeny of BA.5, has been observed with the highest proportion of infections within the Moroccan population (Fig. 1 and Table 3). Furthermore, the subvariants BA.2.12.1, BA.4, and BA.5 have also shown a higher rate of infection, in part due to mutations such as S371F, D405N, and R408S, which could allow these subvariants to evade antibodies [43,48]. Remarkably, in Morocco, the evolution of SARS-CoV-2 has been characterized by substantial genetic diversity, with a particularly marked predominance of the Omicron variant and its subvariants over time. This evolutionary trend has also been observed in several countries during the pandemic [26,49]. In Morocco, Omicron and its subvariants accounted for 59.5% of sequences, while Delta was observed at frequency of 11.30 %. Approximate prevalences of Omicron and Delta variants have been reported elsewhere [26]. Hamzaoui and colleagues reported that in Egypt, 55.3% of sequences were assigned to Omicron and 12.1% to Delta. In addition, Omicron subvariants such as BA.5.2.20 and BA.1 were dominant in Morocco (Fig. 1a), accounting for 11 and 10.1% of sequences, respectively (Table 3). Our observations are consistent with those of Sarkar and colleagues, who reported a predominance of BA.1 in Europe in late 2021 and early 2022 [49]. This trend was also observed in the USA, Brazil, and South Africa, in which BA.1 prevailed over BA.2. Furthermore, in June 2022, a trend of BA.4 and BA.5 subvariants was observed. This observation is consistent with previous studies [26,49]. However, unlike some North African countries, such as Egypt, the BA.5.2(13.75%) and B.1.617.2(8.74%) variants were the most prevalent, while in Algeria, BA.5.2(17.27%) and B.1.617.2(12%) were predominant [26]. Nevertheless, despite the notable spread of Omicron worldwide, some pre-Omicron lineages persisted and were dominant in other countries [26,49]. Notably, in Libya, B.1.525(44.94%) and A (9.55%) predominated; in Tunisia, AY.122(24.23%) and B.1.1.7(21.67%) were dominant; and in Mauritania, B.1.525(31.03%) and AY.34.1(27.58%) were the most dominant [26]. In general, the peaks of predominance and the heterogeneity of SARS-CoV-2 lineages observed in this study and those reported in previous studies highlight the genetic diversity of SARS-CoV-2 during its worldwide spread [26,49].

Furthermore, in Morocco, the BQ.1 lineage (BA.5+R346T+N460K) and one of its sublineages BQ.1.1 (BQ.1+R346T), both descendants of BA.5, which emerged from Botswana/Hong Kong/South Africa, have attracted particular interest and were among the monitored subvariants. These lineages (BQ.1 and BQ.1.1), partially aliased to BA.5.3.1.1.1.1.1.1 were identified in September and November 2022, respectively (File S4). These subvariants stood out by several key mutations, notably R346T, K417N, N440K, K444T, L452R, N460K, F486V, E484A, and N501Y, known for their ability to escape antibodies. In particular, the mutations R346T, K444T, and F486V appear to promote evasion of humoral immune response induced by vaccination [48,50,52]. Furthermore, the mutations L452R, N460K, and R346T observed in the Spike glycoprotein would be correlated with the virus’s ability to bind more efficiently to ACE2 receptors. Consequently, this could increase the virus’s transmissibility or its ability to infect cells. These observations have been reported in previous studies [50,52]. It is worth noting that in Morocco, BQ.1.1 and BQ.1, rapidly reached their epidemic peak in December 2022 (Fig. 1a). Similar epidemiological trends have been described elsewhere, such as in the United States and Europe [53]. While BQ.1 and BQ.1.1 were declining in Morocco towards the end of 2022, new subvariants emerged simultaneously, among which was the XBB (22F) lineage and its progeny (Fig. 6a, Table 4). Unlike what was observed in Europe and the United States, cases of XBB emerged with rapid increases. In contrast, our study revealed sporadic circulation of XBB and its progeny [53]. In this regard, the ancestor (XBB) of these new subvariants was characterized by mutations such as D614G, D405N, E484A, F486S, F490S, G446S, K417N, N440K, N460K, P681H, R408S, S371F, S477N, T376A, T478K, and V445P, all known for their ability to increase transmissibility and evade the immune system [44,54, 55]. Among the descendants of XBB, the GP.2 subvariant of clade 23C (CH.1.1) stood out due to the presence of the key mutation K444T (XBB+K444T), enhancing the virus’s evasive capacity. Furthermore, the mutation G446S observed in XBB and BA.2.75.5 lineage related to BA.2.75 conferred them a resistance to antibodies [50]. Additionally, subvariants such as XBB.1.5 (XBB+F486P), XBB.1.16 (XBB+E180V+F486P), and EG.5.1 (XBB.1+Q52 H + F456L + F486 P / EG.5+Q52 H), harboured mutations that conferred them an evasive capacity against neutralizing antibodies, notably R408S, G446, G339D, D405N, F490S, K417N, and Q498R (Table 4) [55]. It should be noted that EG.5.1 (Eris) from clade 23F, descendant of XBB.1.9.2 (clade 23D), has a mutational profile in the Spike glycoprotein similar to that of XBB.1.5 [55]. However, according to updated data from the WHO on 9 February 2024, these previously mentioned lineages are classified as VOIs currently circulating worldwide [56]. Moreover, subvariants such as XBB.1.9.1 (XBB.1+F486P) and XBB.2.3.11 (XBB+T478R + P521S), derived from the XBB.2.3 lineage (23E) (XBB.1+D253G + F486P + P521S), analysed in this study were also less frequent (Table 4). Nevertheless, these lineages are classified by the WHO as VUMs currently in circulation [56]. Furthermore, this study supports previous observations suggesting that Omicron subvariants such as BA.1.1, BQ.1, BQ.1.1, XBB, and its descendants present distinct characteristics and are associated with variable epidemiological trends worldwide [49]. According to our results, these subvariants which appeared between the end of 2022 and during 2023 (XBB and its derivatives) did not lead to large-scale epidemics, as observed elsewhere [49,57]. This observation could be attributed to the implementation of health safety measures and protocols, and the mass vaccination campaign in Morocco aimed at achieving herd immunity in order to curb the spread of SARS-CoV-2.

In addition to the key mutations observed in the Spike glycoprotein of different lineages of SARS-CoV-2, some mutations such as P13L and R203K/G204R located in the nucleocapsid protein have been identified in notable proportions among the 2274 genomic sequences analysed (Fig. 4a). Therefore, these mutations are known to be associated with increased infectivity, structural stability, and virulence of the virus, which could confer on it a better ability to escape the immune response [58,59]. In general, our analysis revealed a prevalence of 71% of R203K/G204R mutations in our isolates, significantly higher than the proportion of 55% reported by Ahmad and his colleagues [60]. These mutations are observed at high frequency in the omicron and its subvariants, as well as in the Alpha variant and the B.1.1 lineage (Fig. 4a).

However, this study had several limitations. This study included both high and low coverage genomic sequences. Although low-coverage sequences can provide additional information on the genomic diversity of the virus, this can also pose methodological challenges and limitations in the interpretation of results, with possible under-representation of certain viral strains or genome regions. In addition, in-depth documentation of mutations in structural and non-structural proteins could provide a better understanding of the genomic diversity of Moroccan isolates of SARS-CoV-2 and their impact on the dynamics of the virus.

## Conclusion

This study provided a detailed analysis of the genomic epidemiology and genetic diversity of SARS-CoV-2 lineages identified in Morocco during the 3 years of the pandemic, enabling a better understanding of the evolution and phylogenetic relationships among different lineages. Several lineages identified in Morocco were closely related to those observed worldwide, except for lineage B.1.528, before their local spread, highlighting the impact of human mobility on the introduction and spread of these lineages during the pandemic. Viral dynamics in Morocco, characterized by a predominance of Alpha, Delta, Omicron variants, and their subvariants, reflected global trends in their evolution. However, the epidemiological trends of some Delta and Omicron subvariants showed variable patterns compared to those observed in other countries. Additionally, several key mutations identified within the lineages analysed were correlated with variations in transmissibility, pathogenicity and antigenicity, which could have contributed to affecting vaccine efficacy and pandemic management. However, the set-up of the SARS-CoV-2 genomic surveillance consortium in Morocco and vaccination campaigns have contributed to control and reduce infection rates and severe forms of COVID-19, thus mitigating the impact of infections at national level.

## Abbreviations

ACE2: angiotensin converting enzyme 2
COVID-19: coronavirus disease 2019
CT: cytoplasmic domain
FP: fusion peptide
HR1/HR2: heptad repeats 1 and 2
NSP: non-structural protein
NTD: N-terminal domain
RBD: receptor-binding domain
RBM: receptor-binding motif
SARS-CoV-2: severe acute respiratory syndrome coronavirus 2
SNP: single nucleotide polymorphism
TM: transmembrane domain
VOC: variants of concern
VOI: variants of interest
VUM: variants under monitoring
